# Merging microarray data from separate breast cancer studies provides a robust prognostic test

**DOI:** 10.1186/1471-2105-9-125

**Published:** 2008-02-27

**Authors:** Lei Xu, Aik Choon Tan, Raimond L Winslow, Donald Geman

**Affiliations:** 1 The Institute for Computational Medicine and Center for Cardiovascular Bioinformatics and Modeling, Johns Hopkins University, Baltimore, MD 21218, USA; 2 Department of Applied Mathematics and Statistics, Johns Hopkins University, Baltimore, MD 21218, USA

## Abstract

**Background:**

There is an urgent need for new prognostic markers of breast cancer metastases to ensure that newly diagnosed patients receive appropriate therapy. Recent studies have demonstrated the potential value of gene expression signatures in assessing the risk of developing distant metastases. However, due to the small sample sizes of individual studies, the overlap among signatures is almost zero and their predictive power is often limited. Integrating microarray data from multiple studies in order to increase sample size is therefore a promising approach to the development of more robust prognostic tests.

**Results:**

In this study, by using a highly stable data aggregation procedure based on expression comparisons, we have integrated three independent microarray gene expression data sets for breast cancer and identified a structured prognostic signature consisting of 112 genes organized into 80 pair-wise expression comparisons. A classical likelihood ratio test based on these comparisons, essentially weighted voting, achieves 88.6% sensitivity and 54.6% specificity in an independent external test set of 154 samples. The test is highly informative in assessing the risk of developing distant metastases within five years (hazard ratio 9.3 with 95% CI 2.9–29.9).

**Conclusion:**

Rank-based features provide a stable way to integrate patient data from separate microarray studies due to invariance to data normalization, and such features can be combined into a useful predictor of distant metastases in breast cancer within a statistical modeling framework which begins to capture gene-gene interactions. Upon further confirmation on large-scale independent data, such prognostic signatures and tests could provide a powerful tool to guide adjuvant systemic treatment that could greatly reduce the cost of breast cancer treatment, both in terms of toxic side effects and health care expenditures.

## Background

Breast cancer is the most common form of cancer and the second leading cause of cancer death among women in the United States, with an estimated ~213,000 new cases and ~41,000 deaths in 2006 [1]. The main cause of breast cancer death comes from its metastases to distant sites. Early diagnosis and adjuvant systemic therapy (hormone therapy and chemotherapy) substantially reduce the risk of distant metastases. However, adjuvant therapy has serious short- and long-term side effects and involves high medical costs [2]. Therefore, highly accurate prognostic tests are essential to aid clinicians in deciding which patients are at high risk of developing metastases and should receive adjuvant therapy. Currently, the most widely used treatment guidelines, St. Gallen [3] and the US National Institutes of Health (NIH) [2] consensus criteria, assess a patient's risk of distant metastases based on clinical prognostic factors such as tumor size, lymph node status, and histologic grade. These guidelines cannot accurately identify at-risk patients and about 70–80% of patients defined as being at risk by these criteria and receiving adjuvant therapy would have survived without it [4]. In addition, many patients who would be cured by local or regional treatment alone are "over-treated" and suffer toxic side effects of adjuvant therapy unnecessarily. Therefore, there is an urgent need for new prognostic tests to precisely define a patient's risk of developing metastases to ensure that the patient receives appropriate therapy.

The advent of DNA microarray technology provides a powerful tool in various aspects of cancer research. Simultaneous assessment of the expression of thousands of genes in a single experiment could allow better understanding of the complex and heterogeneous molecular properties of breast cancer. Such information may lead to more accurate prognostic signatures for prediction of metastasis risk in breast cancer patients. Over the past few years, a number of studies have identified prognostic gene expression signatures and proposed corresponding prognostic tests based on these genes. In many cases, the prediction of breast cancer outcome is superior to conventional prognostic tests [5-11]. Among these studies, the two largest have attempted to identify gene expression signatures and prognostic tests strongly predictive of distant metastases. van't Veer *et al*. applied a supervised method to identify a 70-gene signature, and a correlation-based test capable of predicting a short interval to distant metastases, in a cohort of 78 young breast cancer patients (<55 years of age) with lymph-node-negative tumors [6]. The test was applied to a cohort of 295 patients with either lymph-node-negative or lymph-node-positive breast tumors [5]. Using a different microarray platform, Wang *et al*. derived a 76-gene prognostic signature from 115 lymph-node-negative patients who had not received adjuvant systemic treatment. The signature could be used to predict distant metastasis within five years in breast cancer patients of all age groups with lymph-node-negative tumors and was subsequently applied to a set of 171 lymph-node-negative patients [7]. These studies have shown that tests based on gene expression signatures would result in a substantial reduction of the number of patients receiving unnecessary adjuvant systemic treatment, thereby preventing over-treatment in a considerable number of breast cancer patients.

The most striking observation when comparing the signatures from different studies is the lack of overlap of signature genes. For instance, in the studies of van't Veer *et al*. and Wang *et al*., despite the similar clinical and statistical designs, there is an overlap of only three genes in the two gene signature lists. These diverse results make it difficult to identify the most predictive genes for breast cancer prognosis. The disagreements in gene signatures may be partly due to the use of different microarray platforms and differences in patient selection, normalization procedures and other experimental choices. Moreover, in a recent study [12], reanalysis of the van't Veer data has shown that the prognostic signature is even strongly influenced by the subset of the patients used for signature selection within a particular study. This observation indicates that given the small number of samples in the training sets, many genes might show what appear to be significant correlations with clinical outcome and the differences among these correlations might be small. Therefore, it is possible to combine genes in many ways to generate different signatures with similar predictive power when validated on internal test sets [12]. Moreover, in general, these prognostic tests are not robust, meaning that they cannot be validated on independent, external data sets [9]. Independent reanalysis on other microarray data sets has shown very similar findings [13]. Given the large numbers of features (~10,000 to 40,000 genes) in microarray data and the relatively small numbers of samples (~100 patients) used in the training set of each study, it is highly possible to accidentally find a set of genes with good predictive power on internal test sets. This is the type of "over-fitting" that is typical when the number of observed variables far exceeds the number of samples. In light of this general "small-sample dilemma" in statistical learning and the particular observations from the two reanalysis studies mentioned above, the disagreements in gene signatures obtained from different data sets are not surprising. We believe that much larger numbers of samples (patients), perhaps thousands, are needed to develop more robust prognostic tests and signatures.

The rapid accumulation of microarray gene expression data suggests that combining microarray data from different studies may be a useful way to increase sample size and diversity. In particular, "meta-analyses" have recently been used to merge different studies in order to develop prognostic gene expression signatures for breast cancer [14,15]. However, effectively integrating microarray data from different studies is not straightforward due to several issues of compatibility, such as differing microarray platforms, experimental protocols and data preprocessing methods. Instead of directly integrating microarray gene expression values, meta-analyses combine results (e.g. *t *statistics) of individual studies to increase statistical power. The major limitation of meta-analyses is that the small sample sizes typical of individual studies, coupled with variation due to differences in study protocols, inevitably degrades the results. Also, deriving separate statistics and then averaging is often less powerful than directly computing statistics from aggregated data.

In contrast to the meta-analysis approach, in which the results of individual studies are combined at an interpretative level, other methods, such as Z-score, Distance Weighted Discrimination (DWD), integrate microarray data from different studies at the expression value level after transforming the expressions to numerically comparable measures [14,16-20]. In general, the procedure involves the following steps. First, a list of genes common to multiple distinct microarray platforms is extracted based on cross-referencing the annotation of each probe set represented on the microarrays. Cross-referencing of expression data is usually achieved using the UniGene database [21]. Next, for each individual data set, numerically comparable quantities are derived from the expression values of genes in the common list by applying specific data transformation and normalization methods. Finally, the newly derived quantities from individual data sets are combined to increase sample size and statistical methods are applied to the combined data to build diagnostic and prognostic signatures. One major limitation of these direct integration methods is that there is still no consensus on how best to perform data transformation and normalization.

In our previous work [22], we proposed a novel method for molecular classification which builds predictors from *relative *expression values, which can be directly applied to integrated microarray data and which generates very simple decision rules. Because this method is based only on the ranks of the expression values within a profile (sample), there is no need to prepare the data for integration, in particular there is no need for data normalization, since ranks are invariant to all types of within-array monotonic preprocessing. This approach to data integration was validated on prostate cancer data [23], resulting in a powerful two-gene diagnostic classifier. It has also been applied recently to differentiating between gastrointestinal stromal tumors and leiomyosarcomas [24]. Here, we extend this method to predict distant metastases in breast cancer, and attempt to overcome the limitations of previous study-specific methods and meta-analyses.

## Results

### Summary

We integrate three independent microarray gene expression data sets to obtain an integrated training set of 358 samples and identify a set of features for predicting distant metastases. All the samples included in this study are from lymph-node-negative patients who have not received adjuvant systemic treatment. Each feature is based on an ordered pair of genes and assumes the value one if the first gene is expressed less than the second gene, and assumes the value zero otherwise. These genes may not all be highly differentially expressed, and one gene in the pair may serve as a "reference" for the other one. Since the features are rank-based, no data normalization is needed before data integration. A classical likelihood ratio test is used to classify patients as either poor-outcome, meaning they are likely to metastasize, or good-outcome, meaning that they are unlikely to develop distant metastases. The choice of features is motivated by achieving the highest possible specificity at an acceptable level of sensitivity, taken here to be 90% in accordance with the St. Gallen and NIH treatment guidelines. The number of features chosen in the prognostic signature, as well as the threshold in the likelihood ratio test (LRT), is optimized with *k*-fold cross-validation on the integrated training set. The optimal feature number is estimated to be 80, corresponding to 112 genes (since some genes appear in more than one feature). The prognostic test based on this signature is validated using an independent microarray data set. Upon further validation on large-scale independent data, the prognostic gene expression signature could support other breast cancer prognostic tests with high enough specificity to help avoid over-treatment of newly diagnosed patients.

### Study data

Four breast cancer microarray data sets are included in this study. Each data set has been downloaded from publicly available gene expression repositories (e.g. Gene Expression Omnibus) or supporting web sites [7,11,25,26]. All four data sets are generated from the same Affymetrix HG-U133A microarray platform. Here, the names of the first authors of individual studies are used as the names of the data sets. Three data sets, Miller (251 patients), Sotiriou (189 patients) and Wang (286 patients), are used as training data and the other one, Pawitan (159 patients), is used as independent test data. The reason for this division into training and test data is that detailed clinical information has been provided for the Miller, Sotiriou and Wang data sets and this information has been used to select specific patients for training, whereas little clinical information is provided for the Pawitan study. For the Miller, Sotiriou and Pawitan studies, because the gene expression data sets provided by them have undergone cross-sample normalization, we have downloaded the raw CEL files and calculated expression values using the Affymetrix GeneChip Operating Software version 1.4. There is an 85-patient overlap between Miller and Sotiriou data sets, so we have excluded the replicate samples from our study. Detailed patient information in each study has been described in the corresponding literature.

Motivated by a recent study [27], we employ the idea of restricting training data to extreme patient samples, which are more informative in identifying a prognostic signature. Extreme patients are either short-term survivors with poor-outcome within a short period or long-term survivors who maintain a good-outcome after a long follow-up time. Specifically, we select patients who developed distant metastases (relapse) within five years as poor-outcome samples and patients who were free of distant metastases (relapse) during the follow-up for a period of at least eight years as good-outcome samples. The sharp contrast between short-term and long-term survivors should identify more informative and reliable genes for a prognostic signature. Only early stage lymph-node-negative patients who had not received adjuvant systemic treatment are included in the training data because adjuvant treatment is likely to modify patient outcome. The selection is irrespective of age, tumor size and other clinical parameters. After applying the above selection criteria, a total of 358 patients are identified from the three training data sets and used to learn a prognostic signature and prognostic test. The numbers of selected patients from each training data set are listed in Table 1.

**Table 1:** Training data sets: lymph-node-negative patients with no adjuvant treatment

**Data Set**	**No. of Patients**	**No. of Good-outcome**	**No. of Poor-outcome**
Miller [25]	106	92	14
Sotiriou [11]	43	30	13
Wang [7]	209	114	95

Total	358	236	122

### A prognostic signature from integrated data

**Figure 1:**
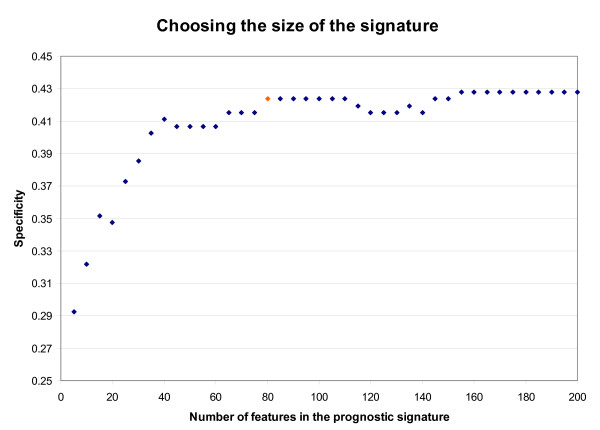
**Choosing size of the signature**. The relationship between the number of features in a prognostic signature and the specificity at 90% sensitivity of the corresponding prognostic test, evaluated by 40-fold cross-validation. We select *m*_*opt *_= 80, the smallest value that achieves roughly maximum specificity at the 90% sensitivity level. The specificity observed on the validation set is in fact higher.

**Figure 2:**
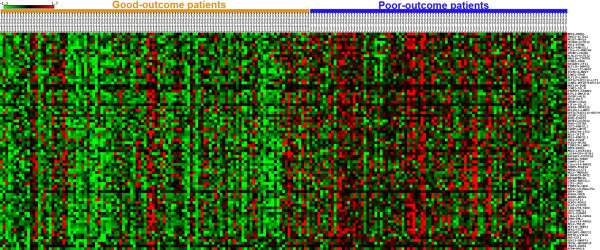
**The heat map of the 80 signature gene pairs**. The Wang data set is used to illustrate the gene expression values of the signature genes. A heat map is generated using the matrix2png software [34]. There are 80 rows corresponding to the 80 gene pairs; the displayed intensities are the differences between the expression values of the two genes in each pair. The expression value for each difference is normalized across the samples to zero mean and one standard deviation (SD) for visualization purposes. Differences with expression levels greater than the mean are colored in red and those below the mean are colored in green. The scale indicates the number of SDs above or below the mean.

We directly merge the three microarray data sets in Table 1, using the 22283 probe sets on Affymetrix HG-U133A microarray, to form an integrated training data set. The integrated data set consists of 122 extreme poor-outcome samples (distant metastases within five years after surgery) and 236 extreme good-outcome samples (free of distant metastases during the follow-up for a period of at least eight years after surgery). Recall that each feature is based on a pair of genes. The integrated training set is used to estimate the relationship between the number *m *of features in a prognostic classifier and the specificity at 90% sensitivity level, evaluated by the 40-fold cross-validation, as described in 'Methods'. The result is plotted in Figure 1. As can be seen, the specificity is nearly constant after about 80 features are included. Our final prognostic signature then consists of the 80 top-ranked features (gene pairs) from the feature list generated from the original integrated training data, using the feature selection and transformation procedures described in 'Methods'. Because some genes appear in more than one feature, the 80 top-ranked gene pairs in our prognostic signature include 112 distinct genes (Table 2). To illustrate the behavior of the 80 features in the signature on the Wang data set (part of the integrated training data), we show the difference in expression between the two genes in each of the 80 gene pairs in the form of a heat map in Figure 2. Distinct patterns of expression differences can be observed for good- and poor-outcome samples.

**Table 2:** Genes in the identified prognostic signature. For each probe set the first column lists the subset of the eighty pairs which contain it. The pairs are ordered from 1 to 80 by their scores.

**Pair Rank**	**Probe Set**	**Gene Symbol**	**Gene Title**
1, 43	91816_f_at	RKHD1	ring finger and KH domain containing 1
1, 6, 73	204641_at	NEK2	NIMA (never in mitosis gene a)-related kinase 2
2	213139_at	SNAI2	snail homolog 2 (Drosophila)
2, 4, 9, 33	212188_at	KCTD12	potassium channel tetramerisation domain containing 12
3	212022_s_at	MKI67	antigen identified by monoclonal antibody Ki-67
3, 61, 80	219716_at	APOL6	apolipoprotein L, 6
4	205264_at	CD3EAP	CD3e molecule, epsilon associated protein
5	206687_s_at	PTPN6	protein tyrosine phosphatase, non-receptor type 6
5, 67	218009_s_at	PRC1	protein regulator of cytokinesis 1
6, 35, 39, 55	219579_at	RAB3IL1	RAB3A interacting protein (rabin3)-like 1
7	221824_s_at	MARCH8	membrane-associated ring finger (C3HC4) 8
7	209574_s_at	C18orf1	chromosome 18 open reading frame 1
8	210199_at	CRYAA	crystallin, alpha A
8, 24, 26, 31	219493_at	SHCBP1	SHC SH2-domain binding protein 1
9	204177_s_at	KLHL20	kelch-like 20 (Drosophila)
10, 34	203010_at	STAT5A	signal transducer and activator of transcription 5A
10	212747_at	ANKS1A	ankyrin repeat and sterile alpha motif domain containing 1A
11, 19, 21	205034_at	CCNE2	cyclin E2
11, 65	217427_s_at	HIRA	HIR histone cell cycle regulation defective homolog A (S. cerevisiae)
12, 46, 54, 74	222077_s_at	RACGAP1	Rac GTPase activating protein 1
12, 62	36545_s_at	SFI1	Sfi1 homolog, spindle assembly associated (yeast)
13, 17, 72	218883_s_at	MLF1IP	MLF1 interacting protein
13	203332_s_at	INPP5D	inositol polyphosphate-5-phosphatase, 145kDa
14, 15	211584_s_at	NPAT	nuclear protein, ataxia-telangiectasia locus
14	219512_at	C20orf172	chromosome 20 open reading frame 172
15	221193_s_at	ZCCHC10	zinc finger, CCHC domain containing 10
16	221521_s_at	GINS2	GINS complex subunit 2 (Psf2 homolog)
16	209671_x_at	TRA@///TRAC	T cell receptor alpha locus///T cell receptor alpha locus
17	208952_s_at	LARP5	La ribonucleoprotein domain family, member 5
18, 30	218726_at	DKFZp762E1312	hypothetical protein DKFZp762E1312
18, 51	211581_x_at	LST1	leukocyte specific transcript 1
19	221273_s_at	DKFZP761H1710	hypothetical protein DKFZp761H1710
20	205395_s_at	MRE11A	MRE11 meiotic recombination 11 homolog A (S. cerevisiae)
20, 59	214973_x_at	IGHD	immunoglobulin heavy constant delta
21, 27	211881_x_at	IGLJ3	immunoglobulin lambda joining 3
22	202602_s_at	HTATSF1	HIV-1 Tat specific factor 1
22	218143_s_at	SCAMP2	secretory carrier membrane protein 2
23	212911_at	DNAJC16	DnaJ (Hsp40) homolog, subfamily C, member 16
23	204817_at	ESPL1	extra spindle poles like 1 (S. cerevisiae)
24	215783_s_at	ALPL	alkaline phosphatase, liver/bone/kidney
25, 38, 39, 44, 52, 71	204825_at	MELK	maternal embryonic leucine zipper kinase
25	213689_x_at	RPL5	Ribosomal protein L5
26	206545_at	CD28	CD28 molecule
27	206364_at	KIF14	kinesin family member 14
28, 60, 61	208079_s_at	AURKA	aurora kinase A
28	214955_at	TMPRSS6	transmembrane protease, serine 6
29	210966_x_at	LARP1	La ribonucleoprotein domain family, member 1
29	218830_at	RPL26L1	ribosomal protein L26-like 1
30	204498_s_at	ADCY9	adenylate cyclase 9
31	206211_at	SELE	selectin E (endothelial adhesion molecule 1)
32, 34, 69	201890_at	RRM2	ribonucleotide reductase M2 polypeptide
32	219298_at	ECHDC3	enoyl Coenzyme A hydratase domain containing 3
33	204847_at	ZBTB11	zinc finger and BTB domain containing 11
35, 62	203214_x_at	CDC2	cell division cycle 2, G1 to S and G2 to M
36	204605_at	CGRRF1	cell growth regulator with ring finger domain 1
36	211251_x_at	NFYC	nuclear transcription factor Y, gamma
37, 65	213008_at	KIAA1794	KIAA1794
37, 73	210042_s_at	CTSZ	cathepsin Z
38	203595_s_at	IFIT5	interferon-induced protein with tetratricopeptide repeats 5
40	221529_s_at	PLVAP	plasmalemma vesicle associated protein
40	202114_at	SNX2	sorting nexin 2
41	211779_x_at	AP2A2	adaptor-related protein complex 2, alpha 2 subunit
41, 63	202324_s_at	ACBD3	acyl-Coenzyme A binding domain containing 3
42, 57	201821_s_at	TIMM17A	translocase of inner mitochondrial membrane 17 homolog A (yeast)
42	201551_s_at	LAMP1	lysosomal-associated membrane protein 1
43	48808_at	DHFR	dihydrofolate reductase
44	211643_x_at	LOC651961	Myosin-reactive immunoglobulin light chain variable region
45	210396_s_at	LOC440354	PI-3-kinase-related kinase SMG-1 pseudogene
45	201070_x_at	SF3B1	splicing factor 3b, subunit 1, 155kDa
46	207391_s_at	PIP5K1A	phosphatidylinositol-4-phosphate 5-kinase, type I, alpha
47	200800_s_at	HSPA1A	heat shock 70 kDa protein 1A
47	201009_s_at	TXNIP	thioredoxin interacting protein
48	203530_s_at	STX4	syntaxin 4
48, 50	218085_at	CHMP5	chromatin modifying protein 5
49, 68, 70	219555_s_at	C16orf60	chromosome 16 open reading frame 60
49	210419_at	BARX2	BarH-like homeobox 2
50	214119_s_at	FKBP1A	FK506 binding protein 1A, 12 kDa
51, 58	203362_s_at	MAD2L1	MAD2 mitotic arrest deficient-like 1 (yeast)
52	218910_at	TMEM16K	transmembrane protein 16K
53	208838_at	KIAA0829	KIAA0829 protein
53	212081_x_at	BAT2	HLA-B associated transcript 2
54	202115_s_at	NOC2L	nucleolar complex associated 2 homolog (S. cerevisiae)
55	209714_s_at	CDKN3	cyclin-dependent kinase inhibitor 3 (CDK2-associated dual specificity phosphatase)
56	205701_at	IPO8	importin 8
56	205063_at	SIP1	survival of motor neuron protein interacting protein 1
57	200918_s_at	SRPR	signal recognition particle receptor ('docking protein')
58	212527_at	D15Wsu75e	DNA segment, Chr 15, Wayne State University 75, expressed
59	204244_s_at	DBF4	DBF4 homolog (S. cerevisiae)
60	214508_x_at	CREM	cAMP responsive element modulator
63	200787_s_at	PEA15	phosphoprotein enriched in astrocytes 15
64	203764_at	DLG7	discs, large homolog 7 (Drosophila)
64	205877_s_at	ZC3H7B	zinc finger CCCH-type containing 7B
66	200848_at	AHCYL1	S-adenosylhomocysteine hydrolase-like 1
66	201091_s_at	CBX3	chromobox homolog 3 (HP1 gamma homolog, Drosophila)
67	64064_at	GIMAP5	GTPase, IMAP family member 5
68	211649_x_at	IGHG1	Immunoglobulin heavy constant gamma 1 (G1m marker)
69	204398_s_at	EML2	echinoderm microtubule associated protein like 2
70	220433_at	PRRG3	proline rich Gla (G-carboxyglutamic acid) 3 (transmembrane)
71	219169_s_at	TFB1M	transcription factor B1, mitochondrial
72	34689_at	TREX1	three prime repair exonuclease 1
74	212604_at	MRPS31	mitochondrial ribosomal protein S31
75	213907_at	EEF1E1	Eukaryotic translation elongation factor 1 epsilon 1
75	209622_at	STK16	serine/threonine kinase 16
76	209716_at	CSF1	colony stimulating factor 1 (macrophage)
76	219575_s_at	PDF	peptide deformylase (mitochondrial)
77	219328_at	DDX31	DEAD (Asp-Glu-Ala-Asp) box polypeptide 31
77	213121_at	SNRP70	small nuclear ribonucleoprotein 70 kDa polypeptide (RNP antigen)
78	218870_at	ARHGAP15	Rho GTPase activating protein 15
78	219105_x_at	ORC6L	origin recognition complex, subunit 6 like (yeast)
79	216510_x_at	IGHA1	immunoglobulin heavy constant alpha 1
79	215207_x_at	YDD19	YDD19 protein
80	219918_s_at	ASPM	asp (abnormal spindle)-like, microcephaly associated (Drosophila)

In order to evaluate the reproducibility of the 112-gene signature, we repeat the same feature selection process with several re-samplings of 300 patients out of the 358 patients in the integrated data set. The average overlap is 39.0%. This is not surprising in view of the still modest sample size and the fact that most of the changes occur in the second half of the ranked list of gene pairs.

### Validation of the prognostic test on independent data

To validate the prognostic test, we compute its sensitivity and specificity on an independent set of samples, the Pawitan data set [26], which consists of 159 primary breast cancer patients. This test set includes both patients with lymph-node-negative tumors and patients with lymph-node-positive tumors, and who had or had not received adjuvant systemic therapy. Following the practice in most of the literature, our objective is to predict the development of distant metastases within five years. Of the 159 patients, 35 patients developed distant metastases (relapse) within five years ("poor-outcome"), and 119 patients were free of distant metastases (no relapse) during the follow-up for a period of at least five years ("good-outcome"). Note that the definition of good-outcome for patients in the validating data is different from the definition in the training data because we have used extreme samples to identify the prognostic signature.

Our prognostic test is the classical likelihood ratio test, determined by assuming that the features are conditionally independent under both classes, namely "poor outcome" (the null hypothesis) and "good outcome" (the alternative hypothesis); see 'Methods'. The LRT reduces to comparing a weighted average of the 80 features to a threshold. The weights depend on the statistics of the individual features under both classes and are estimated from the training data; the threshold is also estimated from the training set, using cross-validation. The LRT built from the prognostic signature achieves a sensitivity of 88.6% (31 out of the 35 poor-outcome samples) and a specificity of 54.6% (65 out of the 119 good-outcome samples) on the 154 samples included in the validating data set. The remaining five patients, who either developed distant metastases after five years or were free of distant metastases with a follow-up period less than five years, are not included in the validating data set. We compute the odds ratio of the prognostic test for developing metastases within five years between the patients in the poor-outcome group and in the good-outcome group as determined by the prognostic test. The prognostic test has a high odds ratio of 9.3 (95% confidence interval: 3.1 – 28.1) with a Fisher's exact test *p*-value < 0.00001. To make the results easier to understand, we have included in the additional files the heat maps of the two-group (good- and poor-outcomes) supervised clusters of the integrated training data and test data for the 112-signature genes (see Additional file 1 and file 2).

It is noteworthy that performance of the LRT on the validation data is actually somewhat *better *than the performance on the training set (which is estimated by cross-validation). Specifically, from Figure 1 (see also 'Methods'), the specificity of the LRT prognostic test is around 43% at approximately 90% sensitivity when estimated from the training data, whereas a specificity of approximately 55% at about the same sensitivity is achieved on the independent validation set.

**Figure 3:**
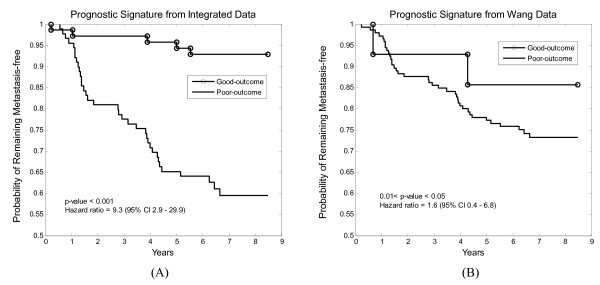
**The Kaplan-Meier analysis**. Kaplan-Meier analysis of the probability of remaining free of distant metastases among 159 Pawitan patients between the good-outcome group and the poor-outcome group. The LRT is based on the integrated data in (A) and the single, Wang data set in (B). CI denotes confidence interval and the *p*-value is calculated by the log-rank test.

To obtain another useful estimate of the clinical outcome, we apply the LRT built from the prognostic signature to all of the 159 samples in the Pawitan data set and calculate the probability of remaining free of distant metastases according to the prognostic signature by using Kaplan-Meier analysis. The Kaplan-Meier curve of the prognostic signature shows a significant difference (*p*-value < 0.001) in the probability of remaining free of distant metastases between the patients in the poor-outcome group and those in the good-outcome groups (Figure 3A). The *p*-value is computed by the use of log-rank test. The Mantel-Cox estimation of hazard ratio for distant metastases within five years in the poor-outcome group as compared to the good-outcome group is 9.3 (95% confidence interval: 2.9 – 29.9, *p*-value < 0.001).

### Comparison of the prognostic signature to study-specific signatures

To evaluate the potential statistical power gained by integrating multiple data sets to increase diversity and sample size, we compare the predictive power of our integrated prognostic signature with each of the three separate study-specific prognostic signatures identified from the three data sets in Table 1. We use exactly the same method we used for the integrated data and each of the resulting three prognostic tests is applied to the same independent test data, the Pawitan data. The results are reported in Table 3. In the case of the Sotiriou data, we do not achieve the targeted sensitivity of at least ninety percent due to the very small sample size; the estimate of the threshold in the LRT does not generalize to the Pawitan test set. For the Miller and Wang data sets, the desired sensitivity is achieved but the specificity is far lower than for the classifier learned from the integrated data set.

**Table 3:** Test results on Pawitan data (154 patients)

**Training Data**	**No. of Patients**	**Sensitivity (%)**	**Specificity (%)**
Sotiriou	43	51.4	47.1
Miller	106	100.0	15.1
Wang	209	94.3	10.1
Integrated	358	88.6	54.6

The Wang data set is the largest. Using 40-fold cross-validation, the optimal feature number of gene pairs for the prognostic signature is *m*_*opt *_= 60. The 94.3% sensitivity on the test set (33 out of the 35 poor-outcome samples) is close to the target of 90%. The specificity of the classifier is 10.1% (12 out of the 119 poor-outcome samples), substantially lower than the classifier based on the integrated training set, albeit at somewhat higher sensitivity. (Indeed, the performance of the prognostic LRT test based on the Wang data alone is barely better than the completely randomized, data-independent procedure which chooses poor-outcome with probability 0.9 and good outcome with probability 0.1, independently from sample to sample.) The odds ratio of this test is 1.9 (95% confidence interval: 0.4 – 8.7, Fisher's exact test p-value = 0.74), and the Kaplan-Meier curve (Figure 3B) shows a less significant difference between the patients in the poor-outcome and good-outcome groups than that of the signature from the integrated data. Finally, the estimated hazard ratio of 1.6 (95% confidence interval: 0.4 – 6.8, 0.01 < p-value < 0.05) is much lower than that of the prognostic test from the integrated data.

These comparisons demonstrate that the prognostic test derived from the integrated data is superior to the prognostic test derived from any of the individual studies and highlight the value of data integration. By integrating several microarray data sets with our rank-based methods, study-specific effects are reduced and more features of breast cancer prognosis are captured.

## Discussion

Using a rank-based method for feature selection, we integrate three independent microarray gene expression data sets of extreme samples and identify a 112-gene breast cancer prognostic signature. The signature is invariant to standard within-array preprocessing and data normalization. All of the patients in the integrated training set had lymph-node-negative tumors and had not received adjuvant systemic treatment, so the identification of the prognostic signature is not subject to potential confounding factors related to lymph node status or systemic treatment. A LRT constructed from the prognostic signature is used to predict whether a breast cancer patient will develop distant metastases within five years after initial treatment. This prognostic test achieves a sensitivity of 88.6% and a specificity of 54.6% on an independent test data set of 154 samples. The test set includes patients who had and who had not received adjuvant systemic treatment, and those with both lymph-node-negative and lymph-node-positive tumors, indicating that our prognostic signature could possibly be applied to all breast cancer patients independently of age, tumor size, tumor grade, lymph mode status, and systemic treatment. It should be pointed out that, somewhat paradoxically, one reason for this ability to generalize is that, as with all machine learning methods, the feature seleciton process is not guided by specific biological knowledge about the underlying processes and pathways.

One motivation for using the LRT is simplicity: under the assumption of independent features, the test statistic is a weighted average of the feature values and the test itself reduces to comparing this average to a fixed threshold. Another motivation stems from the Neyman-Pearson lemma of statistical hypothesis testing [28], which states that the LRT achieves optimal specificity at any given level of sensitivity. However, we cannot claim optimal specificity (at roughly ninety percent sensitivity) for our prognostic test since our LRT is constructed by assuming the 80 binary comparison features are statistically independent in each class, which is likely to be violated in practice due to correlations among the genes and genes appearing in multiple pairs. But this approach does offer a rigorous statistical framework for constructing prognostic tests at a given sensitivity. It also provides a direction towards more powerful procedures. Evidently, increasingly better approximations to the "true" LRT, and hence to optimal specificity, would be obtained by accounting for more and more of the dependency structure among the features. Indeed, accounting for pair-wise correlations alone would be a significant step in this direction.

Comparison with the conventional treatment guidelines (e.g. St. Gallen and NIH) is instructive. While maintaining almost the same level of sensitivity (~90%), our prognostic test achieves a specificity which is well above the 10–30% range of the St. Gallen and NIH targets. This means that our test can spare a significant number of good-outcome patients from unnecessary adjuvant therapy, while ensuring roughly the same percentage of poor-outcome patients receive adjuvant therapy as recommended by the treatment guidelines. Therefore, our prognostic test and signature, if further validated on large-scale independent data, could potentially provide a useful means of guiding adjuvant systemic treatment, reducing cost and improving the quality of patients' lives.

Other strengths of our study, compared with previous ones, are the larger number of homogeneous patients (lymph-node-negative tumors without adjuvant systemic treatment) in the training set, and an external independent test set. In each of the two major breast cancer prognostic studies [6,7], the training and validation data are extracted from the same study group from the same population. More specifically, the entire data set is randomly divided into two pieces, one serving as a training set and the other as a validation or test set. In this case, the training data and the validation data are likely to have similar properties. Therefore, the study-specific prognostic test identified from the training data usually gives over-promising results when assessed using the "internal" validation data. (Similar remarks apply to methods which measure performance using cross validation.) This argument may explain why the two major prognostic signatures, although validated internally with about 90% sensitivity and about 50% specificity, cannot be validated externally with an independent data set [9]. In addition, splitting the original data set into two pieces only aggravates the small-sample problem, as well as producing other sources of bias [12]. In our study, we increase diversity and sample size by integrating several microaray data sets involving patients from different populations. By selecting a homogeneous subgroup of patients and combining data from multiple studies, the derived prognostic test and signature is less sensitive to study-specific factors. An intriguing advantage of inter-study data integration is that it increases the statistical power to capture essential prognostic features which might be masked by study-specific features and the small sample sizes of individual data sets. In this sense, our prognostic test is more robust to inter-study variability and may facilitate external validation.

Comparison of our prognostic signature with the two major signatures of van't Veer *et al*. and Wang *et al*. is not straightforward because of differences in patients, microarray platforms, and algorithms. The study of van't Veer *et al*. uses an Agilent array platform and our study uses an Affymetrix array platform. Only 46 out of the 112 genes in our prognostic signature are present on the Agilent Hu25K array and only 36 of the 70 genes in the van't Veer signature are present on the Affymetrix HG-U133A array. Therefore, we can neither validate the van't Veer prognostic test on our validation data nor validate our test on their data set. There is a three-gene overlap between the van't Veer signature and our signature (CCNE2, ORC6L, and PRC1). Since the data set in Wang *et al*. is included in our training set, we cannot validate our test on that data set. On the other hand, in order to validate the test proposed by Wang *et al*., we need to know the estrogen receptor (ER) status of our test samples because the classification rule based on their signature is depend on ER status, which is absent from our validation data. Again, there is a four-gene overlap between the Wang signature and our signature (AP2A2, CBX3, CCNE2, and MLF1IP). It is noteworthy that the gene CCNE2 is included in all of the three signatures and is reported to be related to breast cancer [29]. CCNE2 could be a potential target for the rational development of new cancer drugs.

Using the program DAVID [30], according to the gene ontology biological process categories, the 112-gene signature is highly enriched in cell cycle (*P*-value = 1.45E-5) and cell division (*P*-value = 5.9E-4). To pinpoint the role of some of the genes in our signature, the cell cycle pathway is displayed in the additional files with our signature genes shown in red (see Additional file 3). These findings demonstrate that deregulation of these pathways has a direct impact on tumor progression and indicate that the 112-gene signature is biologically relevant.

To assess the benefit of data integration, we compared the predictive power of our signature with that of three study-specific signatures identified from the Sotiriou, Miller and Wang data sets using the same LRT procedure. When applied to the same independent test data, our prognostic test consistently outperforms the study-specific tests and the largest study (Wang) in particular, in terms of specificity (54.6% vs. 10.1%) at roughly the same 90% sensitivity level, odds ratio (9.3 vs. 1.9), hazard ratio (9.3 vs. 1.6), and Kaplan-Meier analysis. These findings again suggest a prognostic test derived from a single data set may be over-dedicated and might perform weakly on external data. In contrast, a prognostic test derived from integrated data is more likely to be more robust to study-specific factors and to be validated satisfactorily on external data.

Recently, some studies have shown that combining gene expression data and conventional clinical data (e.g. tumor size, grade, ER status) could lead to improved breast cancer prognosis [31,32]. An approach based on solid statistical principles can accommodate aggregating data of multiple types, e.g., combining gene expression signatures with traditional clinical factors. In this study, due to the lack of clinical information for some of the training samples, we could not incorporate such information into the development of our prognostic test. As clinical information becomes publicly available, it might be combined with the integrated gene expression data to further improve prognosis.

## Conclusion

The opinion expressed in recent studies that gene expression information can be useful in breast cancer prognosis seems to be well-founded. However, due to the small sample sizes relative to the complexity of the entire expression profile, existing methods suffer certain limitations, namely the prevalence of study-specific signatures and difficulties in validating the prognostic tests constructed from these signatures on independent data. Integrating data from multiple studies to obtain more samples appears to be a promising way to overcome these limitations.

We have integrated several gene expression data sets and developed a likelihood ratio test for predicting distant metastases that correctly signals a poor outcome in approximately ninety percent of test cases while maintaining about fifty-five percent specificity for good outcome patients. This well exceeds the St. Gallen and NIH guidelines and compares favorably with the best results previously reported (although not yet validated on external test data). As more and more gene (and protein) expression data is generated and made publicly available, modeling the interactions among genes (and gene products) will become increasingly feasible, and is likely to be crucial in designing prognostic tests which achieve high sensitivity without sacrificing specificity.

## Methods

### Data integration

Recently, our group has developed a family of statistical molecular classification methods based on relative expression reversals [22,33], and applied one variant based on a two-gene classifier to microarray data integration [23]. These methods only use the ranks of gene expression values within each profile and achieve impressive results in both molecular classification and microarray data integration. An important feature of rank-based methods is that they are invariant to monotonic transformations of the expression data within an array, such as those used in most array normalization and other pre-processing methods. This property makes these methods especially useful for combining data from separate studies since the nature of the primary features extracted from the data, namely comparisons of mRNA concentration between pairs of genes, eliminates the need to standardize the data before aggregation. Specifically, the ranks of gene expression values are invariant to monotonic data transformations within each microarray. Consequently, we directly merge gene expression data of the patients from three training data sets in Table 1, using the 22283 probe sets on Affymetrix HG-U133A microarray, to form an integrated training data set of 358 samples. After aggregation, we extract a list of pair-wise comparisons; each of these "features" corresponds to a pair of genes and is assigned the value zero or one depending on the observed ordering of expressions; see the following section. The number of features retained is much smaller than the number of genes on the array. The procedure is now described in more detail.

### Feature selection and transformation

Consider *G *genes whose expression values ***X ***= {*X*_1_, *X*_2_, ..., *X*_*G*_} are measured using a DNA microarray and regarded as random variables. The class label *Y *for each profile ***X ***is a discrete random variable taking on one of two possible values corresponding to the two prognostic states or hypotheses of interest, namely "poor-outcome," denoted *Y *= 1, and "good-outcome," denoted *Y *= 2. The integrated training microarray data represent the observed values of ***X ***and comprise a *G *× *N *matrix ***x ***= [*x*_*gn*_], *g *= 1, 2, ..., *G *and *n *= 1, 2, ..., *N*, where *G *is the number of genes in each profile and *N *is the number of samples (profiles) in the integrated data set. Each column *n *represents a gene expression profile of *G *genes with a class label *y*_*n *_= 1 (poor-outcome) or *y*_*n *_= 2 (good-outcome) for the two-class problem in our study. Among the *N *samples, there are *N*_1 _(respectively, *N*_2_) samples labeled as class 1 (respectively, class 2) with *N *= *N*_1 _+ *N*_2_.

For each pair of genes (*i*, *j*), where *i*, *j *= 1, 2, ..., *G*, *i *≠ *j*, let *P*(*X*_*i *_<*X*_*j*_|*Y *= *k*), *k *= 1,2, denote the conditional probability of the event {*X*_*i *_<*X*_*j*_*} *given *Y *= *k*. We define a score by

(1) *Δ*_*j *_= |*P*(*X*_*i *_<*X*_*j*_|*Y *= 1) - *P*(*X*_*i *_<*X*_*j*_|*Y *= 2)|

and estimate the score of pair (*i*, *j*) based on the training set ***x ***by

(2) $$\Delta_{ij}\approx \left|\frac{N_{ij}^{\left(1\right)}}{N_{1}}-\frac{N_{ij}^{\left(2\right)}}{N_{2}}\right|$$

where

$$\begin{array}{cc} N_{ij}^{\left(k\right)}=\left|\left\{n:1\leq n\leq N,x_{in}<x_{jn},y_{n}=k\right\}\right|, & k=1,2 \end{array}.$$

In other words, the estimated score is simply the absolute difference between the fraction of poor-outcome patients for which gene *i *is expressed less than gene *j *and the same fraction in the good-outcome examples. The feature selection procedure consists of forming a list of gene pairs, sorted from the largest to the smallest according to their scores *Δ*_*ij*_, and selecting the top *M *pairs. The *M *top-ranked gene pairs are then considered to be the most discriminating candidate gene pairs for breast cancer prognosis if only relative expressions are taken into account. During the process, we have transformed the original feature vector ***X ***= {*X*_1_, *X*_2_, ..., *X*_*G*_*}*(*G *= 22283 in this study), each of which assumes scalar values, to a new ordered feature vector ***Z ***= {*Z*_1_, *Z*_2_, ..., *Z*_*M*_*}*(usually, *M *<<*G*), each of which assumes only two values.

Suppose *Z*_*m*_, *m *= 1, 2, ..., *M*, corresponds to the gene pair {*i*, *j*}. For convenience, the ordering (*i*, *j*) will signify which probability in Equation (1) is larger. The reason for this is to facilitate interpretation of the results, as will become apparent. If *P*(*X*_*i *_<*X*_*j*_|*Y = 1*) ≥ *P*(*X*_*i *_<*X*_*j*_|*Y = 2*), as estimated by the fractions in (2), we will write (*i*, *j*) and if *P*(*X*_*i *_<*X*_*j*_|*Y = 1*) <*P*(*X*_*i *_<*X*_*j*_|*Y = 2*) we will denote the pair by (*j*, *i*). The value assumed by *Z*_*m *_is then set to be 1 if we observe *X*_*i *_<*X*_*j *_and set to 0 otherwise, i.e., if we observe *X*_*i *_≥ *X*_*j*_. Of course the same definition is applied to each feature in the training set. In this way, observing *Z*_*m *_= 1 (resp., *Z*_*m *_= 0) represents an indicator of the poor outcome (resp., good outcome) class in the sense that *p*_*m *_= *P*(*Z*_*m *_= 1|*Y *= 1) ≥ *q*_*m *_= *P*(*Z*_*m *_= 1|*Y *= 2). For all the features selected in our signature we in fact have *p*_*m *_> 1/2 > *q*_*m*_.

After this procedure, the original *G *× *N *data matrix is reduced to an *M *× *N *data matrix. The number of distinct genes in a prognostic signature is obviously fewer than *2M*. In our practice, there are always more than *M *distinct genes among the top *M *gene pairs. Given that the numbers of genes in published breast cancer prognostic signatures are mostly fewer than 100, we fix *M *= 200 in this study to maker sure we can identify a prognostic feature signature based on a reasonable number of genes.

### Likelihood ratio test

The classical likelihood ratio test (LRT) is a statistical procedure for distinguishing between two hypotheses, each constraining the distribution of a random vector ***Z ***= {*Z*_1_, *Z*_2_, ..., *Z*_*M*_}. In our case the variables *Z*_*m *_are the simple, binary functions of the gene expression profile defined above.

The LRT is based on the likelihood ratio

$$LR(z)=\frac{P(z|Y=1)}{P(z|Y=2)}$$

where ***z ***= {*z*_1_, *z*_2_, ..., *z*_*M*_*}*are the observed values in a new sample. Notice that ***z ***assumes values in {0, 1}^*M*^, the set of binary strings of length *M*. The LRT chooses hypothesis *Y *= 1 if *LR*(***z***) > *t *and chooses *Y *= 2 otherwise, i.e., if *LR*(***z***) ≤ *t*. The threshold *t *is adjusted to provide a desired tradeoff between type I error and type II error, i.e., between sensitivity and specificity. Choosing *t *small provides high sensitivity at the expense of specificity and choosing *t *large promotes the opposite effect.

#### Naive Bayes Classifier

In the special case in which the random variables *Z*_1_, ..., *Z*_*M *_are binary (as here) and are assumed to be conditionally independent given *Y*, the LRT has a particularly simple form. It reduces to comparing a linear combination of the variables to a threshold. Recall that *p*_*m *_= *P*(*Z*_*m *_= 1|*Y *= 1) and *q*_*m *_= *P*(*Z*_*m *_= 1|*Y *= 2), *m *= 1, 2, ..., *M*. In this case,

$$P(z|Y=1)=\prod_{m=1}^{M}p_{m}^{z_{m}}{\left(1-p_{m}\right)}^{1-z_{m}}$$

and a similar expression holds for *P*(***z***|*Y *= 2) with *p*_*m *_replaced by *q*_*m*_.

It follows that

$$LR(z)=\prod_{m=1}^{M}\frac{p_{m}^{z_{m}}{\left(1-p_{m}\right)}^{1-z_{m}}}{q_{m}^{z_{m}}{\left(1-q_{m}\right)}^{1-z_{m}}}$$

and consequently

$$LLR(z)=\log LR(z)=\sum_{m=1}^{M}z_{m}\log\frac{p_{m}(1-q_{m})}{q_{m}(1-p_{m})}+\sum_{m=1}^{M}\log\frac{1-p_{m}}{1-q_{m}}$$

The LRT then reduces to the form: Choose *Y *= 1 if

(3) $$\sum_{m=1}^{M}\lambda_{m}z_{m}>t'$$

and choose *Y *= 2 otherwise, where

(4) $$\lambda_{m}=\log\frac{p_{m}(1-q_{m})}{q_{m}(1-p_{m})}.$$

Since *p*_*m *_> *q*_*m*_, all these coefficients in Equation (4) are positive and the decision rule in Equation (3) reduces to weighted voting among the pair-wise comparisons: every observed instance of *z*_*m *_= 1 is a vote for the poor outcome class with weight *λ*_*m*_. Moreover, under the two assumptions of i) conditional independence and ii) equal *a priori *class probabilities (i.e., *P*(*Y *= 1) = *P*(*Y *= 2)), this is in fact the Bayes classifier (which is optimal) for the threshold *t *= 0.

#### Sensitivity vs. Specificity

Since our interest lies in high sensitivity at the expense of specificity if necessary, we do not choose *t *= 0. Since we want a very high likelihood of detecting the poor-outcome class, we choose the threshold *t *to achieve high sensitivity, defined to be above 90%. Let *t*_*α *_denote the (largest) threshold achieving sensitivity 1 - α. That is, suppose

$$P(\sum_{m=1}^{M}\lambda_{m}Z_{m}>t_{\alpha }|Y=1)=1-\alpha .$$

(We explain how to estimate *t*_*α *_from the training data in the next sections.) Then, from the Neyman-Pearson lemma, we know that our decision rule achieves the maximum possible specificity at this level of sensitivity. More precisely, this threshold maximizes

$$P(\sum_{m=1}^{M}\lambda_{m}Z_{m}\leq t_{\alpha }|Y=2),$$

which is the probability of choosing good-outcome when in fact good-outcome is the true hypothesis.

Of course this is only a theoretical guarantee and depends very strongly on the conditional independence assumption which is surely violated in practice; indeed, some genes are common to several of the variables *Z*_*m*_. Still, the LRT does provide a framework in which there are clearly stated hypotheses under which specificity can be optimized at a given sensitivity. Moreover, it provides a very simple test and the parameters *p*_*m*_, *q*_*m *_are easily estimated with available sample sizes. Most importantly, the decision procedure dictated by the LRT does indeed work well on independent test data (see 'Results').

### Signature identification and class prediction

In clinical practice, when selecting breast cancer patients for adjuvant systemic therapy, it is of evident importance to limit the number of poor-outcome patients assigned to the good-outcome category. The conventional guidelines (e.g., St. Gallen and NIH) for breast cancer treatment usually call for at least 90% sensitivity and 10–30% specificity. Therefore our objective in selecting the threshold *t *is to maintain high sensitivity (~90%); the specificity is then determined by the sample size and the information content in the features. In order to meet these criteria, we employ *k*-fold cross-validation to estimate the threshold which maximizes specificity at ~90% sensitivity for each signature size for our likelihood ratio test.

The idea is to use *k*-fold cross validation to estimate the sensitivity and specificity for each possible value of *m *= *5, 10, 15, ..., 200*, (the number of features in the LRT) and *t *= *1, 2, ..., 200 *(the threshold in Equation (3)). For each such *m *we then compute the specificity at the largest threshold *t*(*m*) achieving at least 90% sensitivity; this function is plotted in Figure 1. (Obtaining 90% sensitivity can always be achieved by selecting a small enough threshold.) Finally, we then choose the smallest value *m*_*opt *_which (approximately) maximizes specificity; the threshold is then *t*_*opt *_= *t*(*m*_*opt*_). From Figure 1, we see that *m*_*opt *_= *80*.

Specifically, the steps are as follows: 1) Divide the integrated training data set into *k *disjoint subsets of approximately equal sample size; 2) Leave out one subset and combine the other *k*-1 subsets to form a training set; 3) Generate a feature list of *M *gene pairs ranked from most to least discriminating according the score defined in Equation (1) and compute the corresponding binary feature vector of length *M *for every training sample and every left-out sample; 4) Starting from the top five features, sequentially add five features at a time from the ranked list, generating series of 40 feature signatures of sizes *m *= *5, 10, ..., 200*; 5) For each signature in 4), classify the left-out samples using the LRT in Equation (3) for each possible integer threshold *t *= 1, 2, ..., 200 and record the numbers of misclassified poor-outcome and misclassified good-outcome samples; 6) Repeat steps 1)-5) exhaustively for all *k *divisions into training and testing in step 1); 7) Calculate the sensitivity and specificity for the prognostic LRT test for each of the 40 signatures, and keep only the largest threshold for which the sensitivity exceeds 90%. The optimal number of features, *m*_*opt*_, is the smallest number which effectively maximizes specificity.

The final prognostic signature is the *m*_*opt *_top-ranked features (gene pairs) generated from the original integrated training set. The final prognostic test is the LRT with these features and the corresponding threshold *t*_*opt *_= *t*(*m*_*opt*_); this is the classifier which is applied to the validation set and yields the error rates reported in 'Results'.

### Additional statistical analysis

We compute the odds ratio of our prognostic test for developing distant metastases within five years between the patients in the poor-outcome group and good-outcome group as determined by LRT classifier. The *p*-values associated with odds ratios are calculated by Fisher's exact text. We also plot the Kaplan-Meier curve of the signature on the independent test data with *p*-values calculated by log-rank test. The Mantel-Cox estimation of hazard ratio of distant metastases within five years for the signature is also reported. All the statistical analyses are performed using MATLAB.

## Authors' contributions

LX, under the supervision of RLW and DG, collected the microarray data sets and implemented the algorithms; all authors developed the methodology and contributed to the final manuscript.

## Supplementary Material

Additional file 1 Clustering of the training data. Shown is the heat map of the two-group (good- and poor-outcome) supervised clusters of the integrated training data for the 112 signature genes. Those genes which appear in multiple pairs among the 80 gene pairs in the signature will appear multiple times in the heat map. The total number of the rows is 160. Click here for file

Additional file 2 Clustering of the test data. Shown is the heat map of the two-group (good- and poor-outcome) supervised clusters of the test data (Pawitan) for the 112 signature genes. Those genes which appear in multiple pairs among the 80 gene pairs in the signature will appear multiple times in the heat map. The total number of the rows is 160. Click here for file

Additional file 3 The cell cycle pathway. Our signature genes which appear in the cell cycle pathway are shown in red. Click here for file

## References

[B1] Jemal A, Siegel R, Ward E, Murray T, Xu J, Smigal C, Thun MJ (2006). Cancer Statistics, 2006. CA Cancer J Clin.

[B2] Eifel P, Axelson JA, Crowley J, Curran WJ, Deshler A, Fulton S, Hendricks CB, Kemeny M (2001). National Institutes of Health Consensus Development Conference Statement: Adjuvant Therapy for Breast Cancer, November 1-3, 2000. J Natl Cancer Inst.

[B3] Goldhirsch A, Glick JH, Gelber RD, Coates AS, Thurlimann B, Senn HJ, and Panel M (2005). Meeting Highlights: International Expert Consensus on the Primary Therapy of Early Breast Cancer 2005. Ann Oncol.

[B4] Early Breast Cancer Trialists' Collaborative G (1998). Polychemotherapy for early breast cancer: an overview of the randomised trials. The Lancet.

[B5] van de Vijver MJ, He YD, van 't Veer LJ, Dai H, Hart AAM, Voskuil DW, Schreiber GJ, Peterse JL, Roberts C, Marton MJ, Parrish M, Atsma D, Witteveen A, Glas A, Delahaye L, van der Velde T, Bartelink H, Rodenhuis S, Rutgers ET, Friend SH, Bernards R (2002). A Gene-Expression Signature as a Predictor of Survival in Breast Cancer. N Engl J Med.

[B6] van 't Veer LJ, Dai H, van de Vijver MJ, He YD, Hart AAM, Mao M, Peterse HL, van der Kooy K, Marton MJ, Witteveen AT, Schreiber GJ, Kerkhoven RM, Roberts C, Linsley PS, Bernards R, Friend SH (2002). Gene expression profiling predicts clinical outcome of breast cancer. Nature.

[B7] Wang Y, Klijn JG, Zhang Y, Sieuwerts AM, Look MP, Yang F, Talantov D, Timmermans M, Meijer-van Gelder ME, Yu J (2005). Gene-expression profiles to predict distant metastasis of lymph-node-negative primary breast cancer. Lancet.

[B8] Ma XJ, Wang Z, Ryan PD, Isakoff SJ, Barmettler A, Fuller A, Muir B, Mohapatra G, Salunga R, Tuggle JT (2004). A two-gene expression ratio predicts clinical outcome in breast cancer patients treated with tamoxifen. Cancer Cell.

[B9] Naderi A, Teschendorff AE, Barbosa-Morais NL, Pinder SE, Green AR, Powe DG, Robertson JFR, Aparicio S, Ellis IO, Brenton JD, Caldas C (2007). A gene-expression signature to predict survival in breast cancer across independent data sets. Oncogene.

[B10] Chang HY, Nuyten DSA, Sneddon JB, Hastie T, Tibshirani R, Sorlie T, Dai H, He YD, van't Veer LJ, Bartelink H, van de Rijn M, Brown PO, van de Vijver MJ (2005). From The Cover: Robustness, scalability, and integration of a wound-response gene expression signature in predicting breast cancer survival. PNAS.

[B11] Sotiriou C, Wirapati P, Loi S, Harris A, Fox S, Smeds J, Nordgren H, Farmer P, Praz V, Haibe-Kains B, Desmedt C, Larsimont D, Cardoso F, Peterse H, Nuyten D, Buyse M, Van de Vijver MJ, Bergh J, Piccart M, Delorenzi M (2006). Gene Expression Profiling in Breast Cancer: Understanding the Molecular Basis of Histologic Grade To Improve Prognosis. J Natl Cancer Inst.

[B12] Ein-Dor L, Kela I, Getz G, Givol D, Domany E (2005). Outcome signature genes in breast cancer: is there a unique set?. Bioinformatics.

[B13] Michiels S, Koscielny S, Hill C (2005). Prediction of cancer outcome with microarrays: a multiple random validation strategy. The Lancet.

[B14] Shen R, Ghosh D, Chinnaiyan A (2004). Prognostic meta-signature of breast cancer developed by two-stage mixture modeling of microarray data. BMC Genomics.

[B15] Teschendorff A, Naderi A, Barbosa-Morais N, Pinder S, Ellis I, Aparicio S, Brenton J, Caldas C (2006). A consensus prognostic gene expression classifier for ER positive breast cancer. Genome Biology.

[B16] Ramaswamy S, Ross KN, Lander ES, Golub TR (2003). A molecular signature of metastasis in primary solid tumors. Nat Genet.

[B17] Warnat P, Eils R, Brors B (2005). Cross-platform analysis of cancer microarray data improves gene expression based classification of phenotypes. BMC Bioinformatics.

[B18] Benito M, Parker J, Du Q, Wu J, Xiang D, Perou CM, Marron JS (2004). Adjustment of systematic microarray data biases. Bioinformatics.

[B19] Cheadle C, Vawter MP, Freed WJ, Becker KG (2003). Analysis of Microarray Data Using Z Score Transformation. J Mol Diagn.

[B20] Jiang H, Deng Y, Chen HS, Tao L, Sha Q, Chen J, Tsai CJ, Zhang S (2004). Joint analysis of two microarray gene-expression data sets to select lung adenocarcinoma marker genes. BMC Bioinformatics.

[B21] Wheeler DL, Barrett T, Benson DA, Bryant SH, Canese K, Church DM, DiCuccio M, Edgar R, Federhen S, Helmberg W, Kenton DL, Khovayko O, Lipman DJ, Madden TL, Maglott DR, Ostell J, Pontius JU, Pruitt KD, Schuler GD, Schriml LM, Sequeira E, Sherry ST, Sirotkin K, Starchenko G, Suzek TO, Tatusov R, Tatusova TA, Wagner L, Yaschenko E (2005). Database resources of the National Center for Biotechnology Information. Nucleic Acids Res.

[B22] Geman D, d'Avignon C, Naiman DQ, Winslow RL (2004). Classifying gene expression profiles from pairwise mRNA comparison. Statistical Applications in Genetics and Molecular Biology.

[B23] Xu L, Tan AC, Naiman DQ, Geman D, Winslow RL (2005). Robust prostate cancer marker genes emerge from direct integration of inter-study microarray data. Bioinformatics.

[B24] Price ND, Trent J, El-Naggar AK, Cogdell D, Taylor E, Hunt KK, Pollock RE, Hood L, Shmulevich I, Zhang W (2007). Highly accurate two-gene classifier for differentiating gastrointestinal stromal tumors and leiomyosarcomas. PNAS.

[B25] Miller LD, Smeds J, George J, Vega VB, Vergara L, Ploner A, Pawitan Y, Hall P, Klaar S, Liu ET, Bergh J (2005). From The Cover: An expression signature for p53 status in human breast cancer predicts mutation status, transcriptional effects, and patient survival. PNAS.

[B26] Pawitan Y, Bjohle J, Amler L, Borg AL, Egyhazi S, Hall P, Han X, Holmberg L, Huang F, Klaar S, Liu E, Miller L, Nordgren H, Ploner A, Sandelin K, Shaw P, Smeds J, Skoog L, Wedren S, Bergh J (2005). Gene expression profiling spares early breast cancer patients from adjuvant therapy: derived and validated in two population-based cohorts. Breast Cancer Research.

[B27] Liu H, Li J, Wong L (2005). Use of extreme patient samples for outcome prediction from gene expression data. Bioinformatics.

[B28] Doksum KA, Bickel PJ (2006). Mathematical statistics : basic ideas and selected topics.

[B29] Payton M, Scully S, Chung G, Coats S (2002). Deregulation of cyclin E2 expression and associated kinase activity in primary breast tumors. Oncogene.

[B30] Dennis G, Sherman B, Hosack D, Yang J, Gao W, Lane HC, Lempicki R (2003). DAVID: Database for Annotation, Visualization, and Integrated Discovery. Genome Biology.

[B31] Pittman J, Huang E, Dressman H, Horng CF, Cheng SH, Tsou MH, Chen CM, Bild A, Iversen ES, Huang AT, Nevins JR, West M (2004). Integrated modeling of clinical and gene expression information for personalized prediction of disease outcomes. PNAS.

[B32] Sun Y, Goodison S, Li J, Liu L, Farmerie W (2007). Improved breast cancer prognosis through the combination of clinical and genetic markers. Bioinformatics.

[B33] Tan AC, Naiman DQ, Xu L, Winslow RL, Geman D (2005). Simple decision rules for classifying human cancers from gene expression profiles. Bioinformatics.

[B34] Pavlidis P, Noble WS (2003). Matrix2png: a utility for visualizing matrix data. Bioinformatics.

