# Supplementary figures and images for: Merging microarray data from separate breast cancer studies provides a robust prognostic test
Source: BMC Bioinformatics. 2008 Feb 27;9:125. doi: 10.1186/1471-2105-9-125 (PMC2409450; doi:10.1186/1471-2105-9-125)

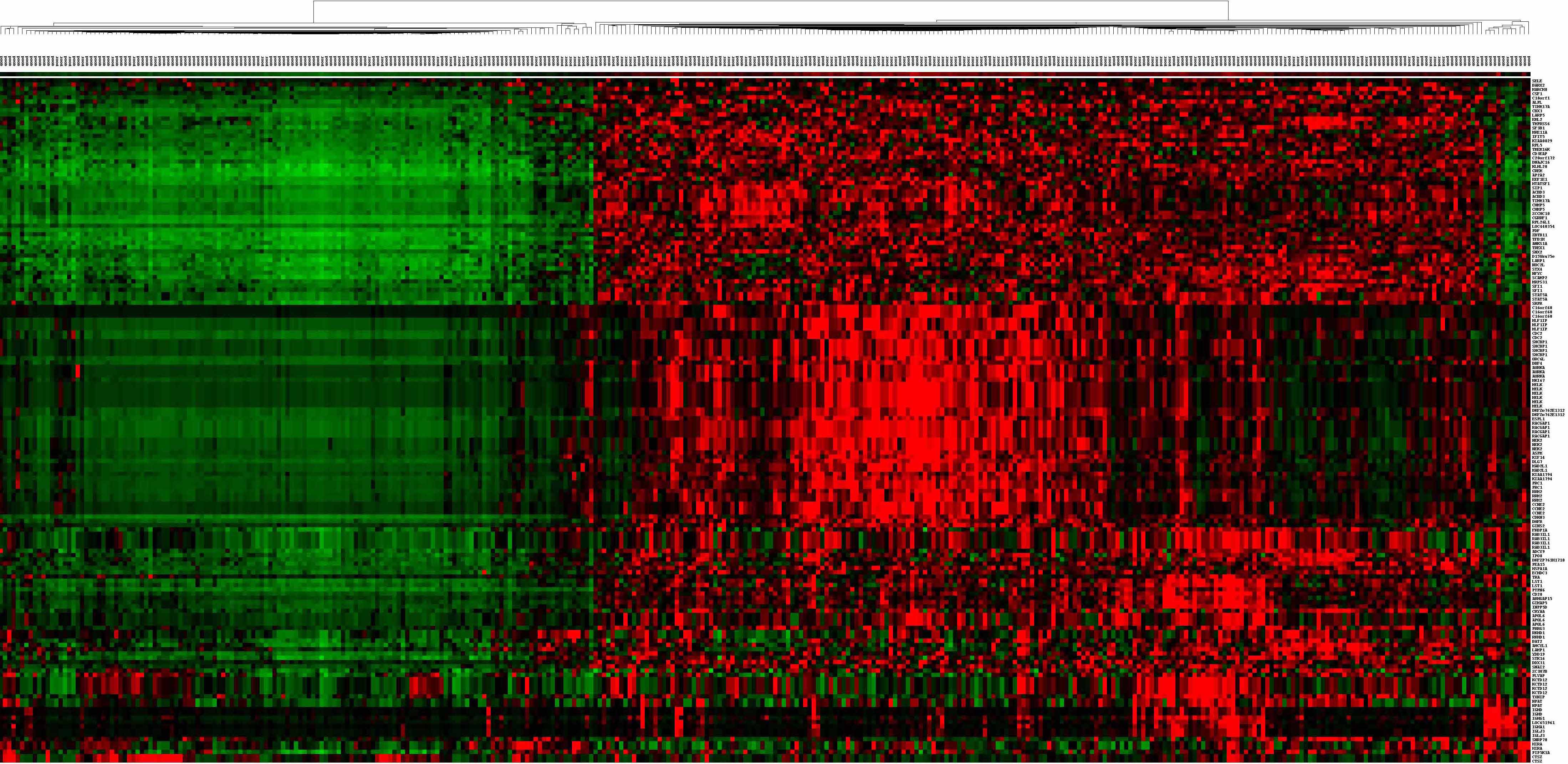

Supplement: Additional file 1 — Clustering of the training data. Shown is the heat map of the two-group (good- and poor-outcome) supervised clusters of the integrated training data for the 112 signature genes. Those genes which appear in multiple pairs among the 80 gene pairs in the signature will appear multiple times in the heat map. The total number of the rows is 160. [file 1471-2105-9-125-S1.jpeg]

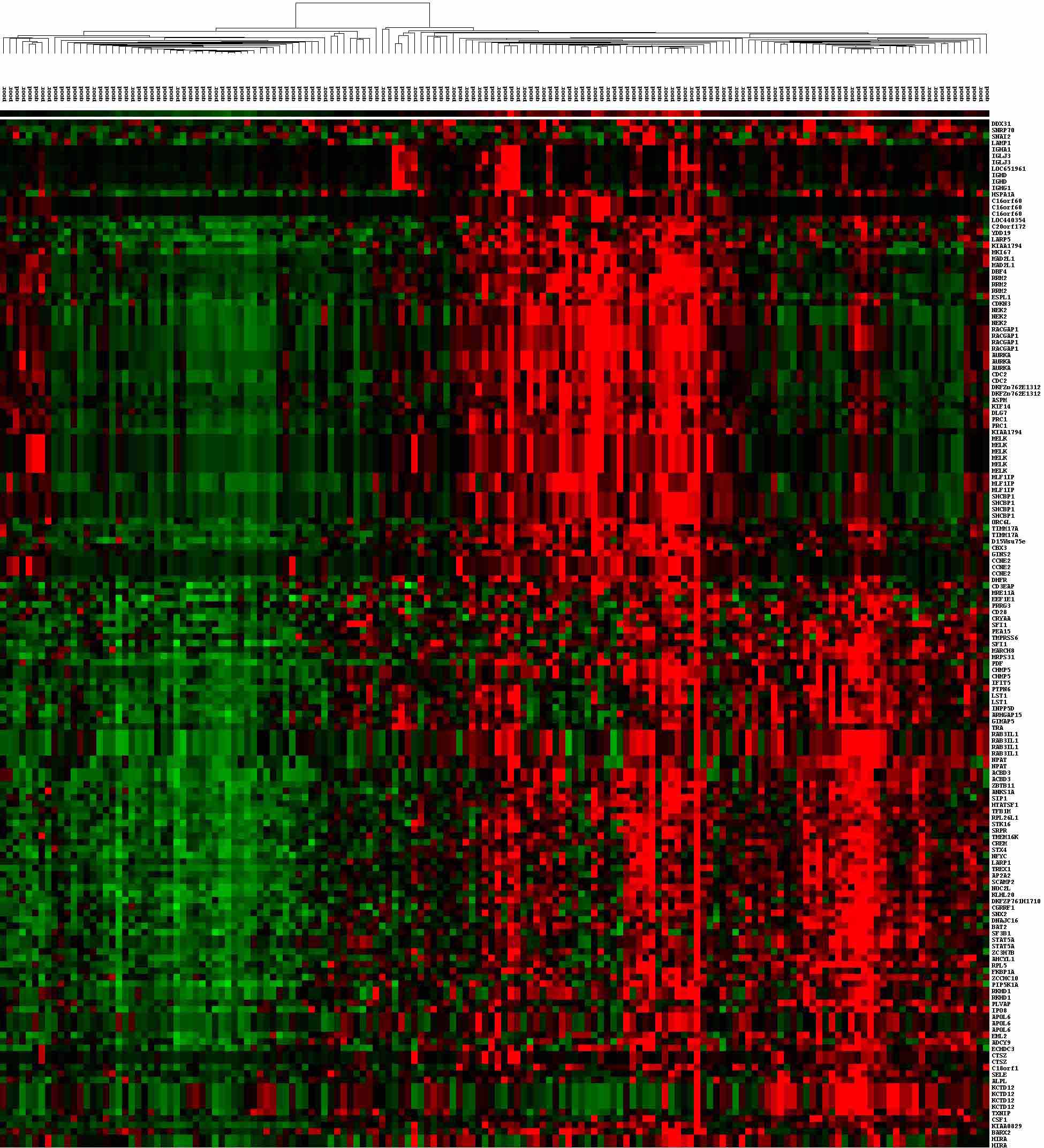

Supplement: Additional file 2 — Clustering of the test data. Shown is the heat map of the two-group (good- and poor-outcome) supervised clusters of the test data (Pawitan) for the 112 signature genes. Those genes which appear in multiple pairs among the 80 gene pairs in the signature will appear multiple times in the heat map. The total number of the rows is 160. [file 1471-2105-9-125-S2.jpeg]

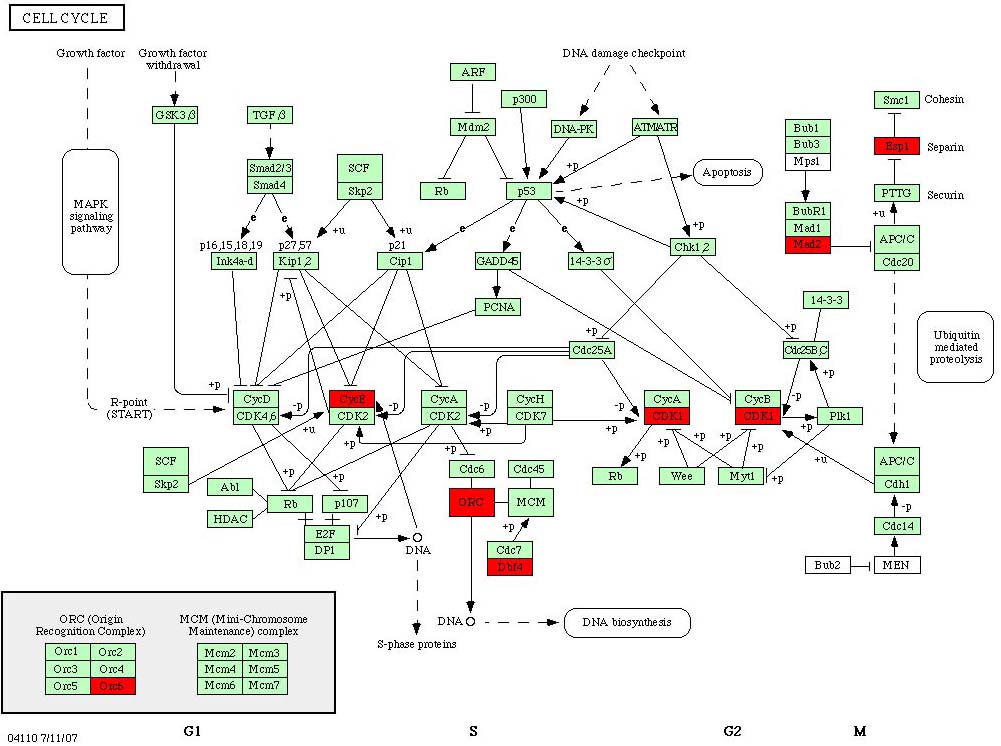

Supplement: Additional file 3 — The cell cycle pathway. Our signature genes which appear in the cell cycle pathway are shown in red. [file 1471-2105-9-125-S3.jpeg]
